# Acquisition of Data on Kinematic Responses to Unpredictable Gait Perturbations: Collection and Quality Assurance of Data for Use in Machine Learning Algorithms for (Near-)Fall Detection

**DOI:** 10.3390/s24165381

**Published:** 2024-08-20

**Authors:** Moritz Schneider, Kevin Reich, Ulrich Hartmann, Ingo Hermanns, Mirko Kaufmann, Annette Kluge, Armin Fiedler, Udo Frese, Rolf Ellegast

**Affiliations:** 1 Institute for Occupational Safety and Health of the German Social Accident Insurance (IFA), 53757 Sankt Augustin, Germany; 2 RheinAhrCampus, Koblenz University of Applied Sciences, 53424 Remagen, Germany; 3 Chair of Work, Organisational & Business Psychology, Ruhr University Bochum, 44801 Bochum, Germany; 4 German Research Center for Artificial Intelligence (DFKI), 28359 Bremen, Germany

**Keywords:** slip, trip, fall, kinematic data, near-fall, machine learning

## Abstract

Slip, trip, and fall (STF) accidents cause high rates of absence from work in many companies. During the 2022 reporting period, the German Social Accident Insurance recorded 165,420 STF accidents, of which 12 were fatal and 2485 led to disability pensions. Particularly in the traffic, transport and logistics sector, STF accidents are the most frequently reported occupational accidents. Therefore, an accurate detection of near-falls is critical to improve worker safety. Efficient detection algorithms are essential for this, but their performance heavily depends on large, well-curated datasets. However, there are drawbacks to current datasets, including small sample sizes, an emphasis on older demographics, and a reliance on simulated rather than real data. In this paper we report the collection of a standardised kinematic STF dataset from real-world STF events affecting parcel delivery workers and steelworkers. We further discuss the use of the data to evaluate dynamic stability control during locomotion for machine learning and build a standardised database. We present the data collection, discuss the classification of the data, present the totality of the data statistically, and compare it with existing databases. A significant research gap is the limited number of participants and focus on older populations in previous studies, as well as the reliance on simulated rather than real-world data. Our study addresses these gaps by providing a larger dataset of real-world STF events from a working population with physically demanding jobs. The population studied included 110 participants, consisting of 55 parcel delivery drivers and 55 steelworkers, both male and female, aged between 19 and 63 years. This diverse participant base allows for a more comprehensive understanding of STF incidents in different working environments.

## 1. Introduction

Slips, trips, and falls (STFs) account for one in five workplace accidents [1]. Such accidents pose a significant challenge to various industries and result in significant periods of absence from work [2,3]. The records of the German Social Accident Insurance (DGUV) Institutions show that these accidents have consistently topped the list of documented occupational accidents year after year, particularly in the transport, logistics and traffic sectors [4,5]. In 2022, the DGUV reported a total of 165,420 STF incidents; 12 of these were fatal, and 2485 disability pensions were paid [6]. Of these incidents, 32% occurred in the commercial sector, including production facilities, workshops, and loading areas, and 16% in generally accessible public and outdoor areas, such as footpaths, car parks, and waiting areas [6]. Factors that reduce surface friction, such as oil spills, moisture, polished surfaces, or meteorological conditions such as rain, snow, or ice, increase the risk of slipping. In addition, uneven surfaces such as steps, sloping surfaces, or raised edges potentially lead to tripping, twisting, or missteps [3]. Certain occupational groups, particularly those in the steel industry, company fire brigades, general firefighters [7], and workers in the public domain such as postal and parcel delivery workers [8], are particularly susceptible to these hazards. The number of STF incidents in these industries has been consistent for many years (as shown by numerous DGUV reports [6,9]), necessitating research into new STF prevention strategies. Since near-falls are predictors for falls [10,11,12], systems to detect them can automatically be used to identify the risks of STF accidents early and then help prevent them.

The development of these detection systems, like machine learning techniques, requires large, diverse, and standardised datasets.

Current datasets used for STF prevention often have significant limitations, impacting their practical application. These databases are often based on elderly populations and have limited sample numbers, which makes them inaccurately reflect the physically demanding nature of many industries. Furthermore, a large portion of the data that are now available is simulated or retrospective, which may not accurately represent real-world situations and may result in less successful preventative interventions. 

The aim of this paper was to generate and analyse standardised kinematic data from STF incidents involving parcel delivery drivers and steelworkers. This study aimed to fill research gaps by providing a larger, more diverse dataset and focusing on a working population in physically demanding jobs. The data will support machine learning applications for accurate near-fall detection, identifying high-risk individuals and enabling targeted fall prevention interventions to improve workplace safety.

### 1.1. State-of-the-Art STF Prevention

Current methodologies support several preventive strategies for reducing the number of STF incidents based on the hierarchy of measures in the so-called STOP model [13,14,15]. Substitution (S) refers to the replacement of potentially dangerous components with alternatives, such as replacing a smooth surface with one with more traction. Technical protective measures (T) include removing items that increase the risk of slipping or installing suitable floor coverings. Organisational protective measures (O) include, e.g., strategic planning of maintenance and renovation projects, and efficient work schedule management. Personal protective measures (P) encompass safety courses, physical training programs, personal protective equipment (helmets, footwear), and personalised safety measures. Protective strategies with the greatest potential impact should be given priority [13]. Business owners or employers are the main target audience for the structured approach of the STOP model, which directs their strategies for managing the risks associated with STFs. A number of international and national organisations like the European Agency for Safety and Health at Work (EU-OSHA), the Health and Safety Executive (HSE), or the DGUV offer workers, companies, and institutions information and guidelines to improve workplace safety and help them follow national occupational safety and health and accident prevention regulations with the goal of reducing (STF) incidents [9,16,17]. The STF incident pattern is influenced by a range of organisational, personal, and technical factors [18,19]. The implementation of the STOP framework improves workplace safety with regards to STF incidents. Certain external variables, however, put newspaper, mail, and parcel couriers at risk. These include traffic, weather (such as rain or snow), and factors outside the organisation’s control (such as the particulars of delivery routes, roads, pavements, driveways, and stairways within buildings). A thorough analysis of STF incidents involving postal workers [20] has shown snowy or icy conditions to be the primary cause in a sizable majority (70%) of the incidents. Three quarters of these falls occurred in the early morning hours (7 am to 9 am), with a significant proportion occurring during the winter months (November to February). Several factors contributed to half of these accidents, including smooth surfaces, insufficient shoe traction, and efficiency-driven actions such as simultaneously walking and preparing mail for later deliveries [20,21] or taking shortcuts [8]. Practical preventive actions addressing such scenarios are outlined in DGUV Information 208-035 (2020) [22]. The focus of the ENTRAPon research project (development of additional training elements to prevent tripping, slipping, and falling accidents), in the context of which the presented measurement data were collected in 2023, lies on high-risk areas (large outdoor areas, private or public premises). Substitution and technical preventive measures against STF incidents are limited in these areas. The approach proposed in this paper includes training to enhance hazard recognition and perturbation-based gait training to enhance postural control mechanisms.

### 1.2. Existing Slip, Trip, and Fall Databases

Approaches to automatic fall detection can be classified in multiple ways, for example, based on which phase of the fall is being detected [23], whether certain data attributes such as acceleration are used [24], or the characteristics of the devices used to capture data [25,26,27]. This diversity of fall detection approaches is accompanied by a wide number of datasets. Igual and colleagues differentiate between context-aware approaches that use data captured by systems deployed in the environment and those using data from wearable devices [26]. Datasets for context-aware systems may consist of recordings by cameras, such as the multi-visual modality fall detection dataset [28] or the multiple cameras fall dataset [29]. Data captured from microphones [30] and pressure data from smart floors [31] also fall into this category. However, these systems are immobile and, therefore, cannot be deployed outdoors, and even in static environments a highly redundant system is needed to achieve appropriate coverage due to elements subject to static and dynamic occlusion [32].

Datasets from automatic approaches to fall detection employing wearable devices are most often recorded by inertial measurement units (IMUs) containing accelerometers, gyroscopes, and magnetometers [26,33]. Devices for measuring heart rate variability, electrocardiograms, and pulse oximetry are also possible [33]. Prominent examples of the wearable device category include the SisFall [34] and Umafall [35] datasets, the former employing one hip-worn sensor for accelerometer and gyroscope data, and the latter five body-worn sensors for accelerometer, gyroscope, and magnetometer data. Approaches that can be assigned to multiple categories are also found. These include the UP-Fall dataset, in which 17 participants were recorded performing activities of daily living (ADLs) and falls by means of wearable sensors and context-aware devices [36].

While the field of actual falls has been frequently studied, studies of near-falls are comparatively rare. A *near-fall* is defined as an incident in which an individual trips, slips, or missteps but is able to regain their balance and keep their feet on the ground. Physical, cognitive, and environmental factors are just some of the many factors contributing to this complex phenomenon [37,38].

While the complexity of near-falls makes studying them challenging, it is still a worthwhile endeavour, since they can indicate whether a person is at a higher risk of falling [10,11,12]. Coupled with the greater incidence of near-falls than falls [12,39,40,41], they can act as early predictors and permit the timely preparation of safety measures. 

Previous research datasets of near-falls include work by Pijnappels et al. [42], who compared the recovery phase of 12 younger and 11 older participants after having tripped over an unanticipated obstacle, and by Wang et al. [12], who researched the automatic detection of near-falls of elderly participants by equipping 34 participants aged over 60 years with a three-axis accelerometer and a full-body marker set and then using sliding panels to induce slips. In our work, we built on these ideas and recorded participants tripping and slipping in a similar way, adding missteps as a potential cause of near-falls.

For a more comprehensive overview of previous work on near-fall datasets recorded with body-worn sensors, we recommend Pang et al. [43]. Among other observations, their findings show that most studies were performed on datasets with 40 or often fewer participants. Only Karel et al. recorded data from 91 participants [37]. Table 1 puts this work in the context of more recent near-fall datasets recorded with wearable devices. It shows that the number of participants in this field remains small.

Since the elderly are at a greater risk of falling than young adults, most of the work discussed above focuses on that age group. Even Choi et al. [38] and Rahemtulla et al. [44] whose participants’ average age was 27.6 and 22–33 years, respectively, conducted their study in the context of fall detection for the elderly. Wang et al. [45] is, to our knowledge, the only study analysing the near-fall events of workers in which the focus lies on preventing workplace accidents. However, this study was also limited to only 10 participants and the near-falls were simulated. Consequently, the falls were not executed spontaneously at an unpredictable moment for the participants, leading to data that inadequately reflect the biomechanical dynamics inherent in genuine falls and near-falls. This problem is found in most fall and near-fall datasets [46], and is one we avoid by inducing actual near-falls in our study.

Our research in this field sought to build on the current findings by addressing important issues. Primarily, the scope for classifying near-falls according to type and intensity by means of kinematic data from IMUs was explored; this may be enhanced by incorporating traditional biomechanical metrics. Furthermore, the scope for using machine learning (ML) to identify near-falls caused by difficult motor tasks was assessed. The foundational dataset needed for ML development was provided in this article.

### 1.3. Research Objective

Most existing near-fall detection techniques employ either ML or single or multi-level threshold-based strategies [33,41,47,48]. The accuracy of fall detection algorithms is evaluated by the means of several metrics. These include the area under the receiver operating characteristic (ROC) curve, positive predictive value (the percentage of correctly identified falls), negative predictive value (the percentage of correctly identified non-falls), specificity (accurate identification of non-falls), and sensitivity (accurate identification of true falls). 

A comparison of machine learning approaches and threshold-based (TH) methods reveals that ML has better false positive rates (ML: 0.6–5.1% vs. TH: 5–10.9%), a wider area under the ROC curve (ML: 0.93–0.99 vs. TH: 0.83–0.96), and superior sensitivities (ML: 84.6–99.8% vs. TH: 72.9–99.7%) [41,48,49,50,51]. Prior studies have proven that TH-centric fall detection mechanisms exhibit higher false positive rates when exposed to near-falls and routine daily activities [49]. Furthermore, because simulated cases have different fall phases than actual fall signals, thresholds—which are usually calibrated using simulated fall data—are often out of alignment with actual fall signals [48].

The selection of sensors, their sampling rates, the participants’ various characteristics, and the type of movements all affect the success of ML-based fall detection. Although machine learning shows promising detection capabilities, algorithmic transferability to various configurations is still a challenge. Considering the scarcity of actual falls, specificity becomes an essential factor.

TH strategies are straightforward and require little computing power, but are not very general, especially when applied to real near-falls. Conversely, ML techniques, despite being heavily dependent on their training datasets and requiring substantial processing power, yield better outcomes for actual near-falls. In terms of accuracy and reliability, they often outperform TH algorithms, owing to their increased sensitivity and decreased false positive rates. However, comprehensive validation and additional investigation under various circumstances require substantial research. In order to advance these investigations, this work presents the recording and preparation of a standardised kinematic dataset. Furthermore, this study seeks to define the baseline using standard approaches. The specific objectives are clearly outlined as follows: first, to compile essential kinematic parameters to describe near-fall detection; second, to analyse detection on a practical yet standardised dataset with the goal of quantifying near-fall slip rate detection and deriving quantitative measures for the intensity of the incidents; and third, to describe the starting point of a dataset for ML, which we expect to quantify improvements in detection.

## 2. Materials and Methods

### 2.1. Experimental Setup and Protocol

A cohort of 110 workers were enrolled in the ENTRAPon research project; this cohort consisted of 55 workers from the steel industry and the postal delivery industry, respectively. Data were collected in three phases. Of the 110 participants at T1, only 109 returned at T2 and 62 remained at T3. All participants took part voluntarily and were given written information explaining the purpose of the study. The research was conducted with the approval of an ethics committee. According to the eligibility criteria, only people in good health and able to perform the tasks assigned without difficulty were included. Participants with neurological or physical limitations, possibly due to previous musculoskeletal disorders or injuries, were excluded from the study.

**Figure 1:**
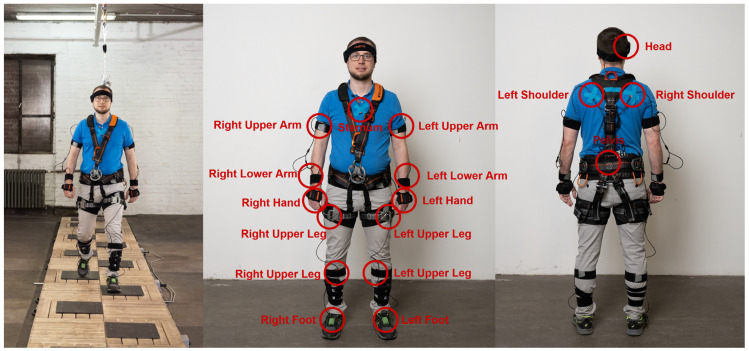
On the left side, one can see the set-up of a slip, trip, and misstep parkour, including an overhead safety system (ceiling) at one cooperation partner’s premises [49]. On the right side, the 17 Xsens Link sensor locations are highlighted.

Participants in the ENTRAPon study negotiated an STF course which was previously described in [49]. In brief, the course consists of a 15 m walkway with 18 integrated STF elements causing unforeseen perturbations, which are divided into three categories: (1) missteps, (2) tripping, and (3) slipping. Black stepping tiles are integrated into the walkway at 70 cm intervals. See Figure 1.

A specially designed tile, mounted on hidden rails with linear bearings, can slide forward by up to 14 cm to simulate slip incidents. A 19 cm board that rises suddenly off the walkway surface in the middle of the swing phase during walking is used to simulate tripping. A foam block that compresses from 11 cm to approximately 5 cm when pressure is applied, causes an unexpected change in height, thereby simulating missteps. The elements were placed within the walkway devoid of a predetermined arrangement, seamlessly integrating into the walkway design. This design ensured that participants were unaware of the exact location or type of perturbation they were to experience. Furthermore, participants were positioned facing away from the walkway before each trial, making it impossible for them to identify the location of potential hazards. 

Each participant was strapped into a harness, attached to an overhead rail for safety, and fitted with shin guards for added protection. Participants were fitted with a full-body measurement suit containing 17 IMU sensors purchased from Xsens Link (Enschede, The Netherlands), which are widely recognised for their effectiveness in assessing gait stability and performing human movement analysis, during both the training and transfer test phases [41,52,53]. This extensive instrumentation enabled participants’ movements in response to the various perturbations presented by the STF course to be precisely recorded and analysed. The IMUs consist of accelerometers, gyrometers, and magnetometers, and data were recorded at a sampling rate of 120 Hz [54].

In accordance with the Xsens guidelines, the study began with the careful measurement of the participants’ anthropometric data like the head height, length of upper extremities and lower extremities, foot height and length, hip depth, etc., and sensor calibration to ensure proper functionality of the Xsens Suit and the CUELA data analysis software [55].

Once the sensors and harness had been attached to the fall arrest device, the participants were positioned at the start of the course. They were instructed to walk at their normal pace while navigating the entire course in each direction. They completed at least three dummy runs (i.e., without perturbations) to ensure that they were comfortable and familiar with the black tile spacing. After this period of familiarisation, the participants were informed that they might now encounter unexpected perturbations during their walk. To ensure genuine, i.e., unexpected reactions, details of the perturbations—such as their timing, location, or nature—were deliberately kept confidential. Participants were also required to stop at the beginning and end of the course, and to begin walking only when given clear instructions to do so. 

Participants had to complete the course successively at two different self-selected walking speeds: (1) at their preferred ‘normal’ walking speed and (2) at a faster walking speed. In each of these walking sessions, the participants experienced each of the three unique perturbations once in varying sequences, namely slipping, tripping, and misstepping. After each incident, they completed the course at least once without an incident. This methodical approach guaranteed thorough exposure to all possible risks, while yielding authentic reactive responses in a variety of walking environments.

During the recording of trials, a marker was automatically placed at the point in time at which a hazard was activated. This marker was then double-checked by a manual review of the data in the Xsens software (MVN Analyse Version 2022.0.2) and in the WIDAAN analysis software [55] to ensure proper marker placement of the STF events.

### 2.2. Data Quality

The concept of data quality has traditionally been defined as fitness for use [56]. In the context of this work, this implies analysing the data’s suitability for future machine learning applications. A review of the literature on data quality revealed a number of key indicators that can be used to assess the quality of a dataset. These include checks for completeness, balance of classes, accuracy of trial labelling, and overall consistency [57,58]. To operationalise completeness for the data we recorded in a laboratory setting, we used the more concrete definition given by Ballou and Pazer: “All values for a certain variable are recorded” [59]. Two levels of completeness are considered: trial-level and sensor-level. Trial-level completeness entails verifying that the requisite number of trial recordings from each participant has been obtained for all three recording days, in accordance with the specifications outlined in the experimental setup. Sensor-level completeness entails confirming the availability of data from each sensor for each frame and the presence of all ergonomic metrics for each trial.

The relative size of classes within the dataset is pivotal for training machine learning classifiers [60]. Our analysis will address potential class imbalances, which may arise due to participants opting out of certain conditions, by calculating the proportion of each STF type (slipping, tripping, misstepping) within the dataset. 

The accurate labelling of trials is significant in the field of machine learning. The objective was to ascertain the precision of the class labels ascribed to the data samples. Each trial was manually labelled in accordance with the activated hazard, with labels (0 s and 1 s) indicating the onset and recovery of an STF event. Although the labels and STF event timing were initially assigned automatically, each instance was subsequently reviewed manually using 3D sensor data mapping, and any necessary corrections were made to the timing. To further validate the accuracy of the labels, three independent reviewers examined the same random sample of 100 trials and compared the assigned labels with the corresponding video recordings.

Finally, we ensured that the sampling rate was consistent throughout the entire dataset, which was a crucial step for maintaining the integrity of time-series data, which is essential for accurate machine learning model training and validation. Furthermore, we confirmed that all data were presented in a consistent format and that each file could be accessed and processed correctly. Although these issues were not explicitly addressed, the comprehensive completeness checks conducted implicitly covered them, thereby ensuring a reliable and uniform dataset.

### 2.3. Data Classification Approach

Our near-fall detection methodology involves two main steps: data collection and data classification. In the data collection phase, we used wearable sensors to collect multivariate time series data from workers as they performed the previously described task on the course. The acquired data can be used to identify near-fall incidents. The data will then be classified according to a scheme that we are currently developing. The scheme will distinguish between normal activity (no fall), near-falls (with and without subsequent fall) and falls. It also categorises near-falls according to their intensity (mild and severe) and the type of STF incident (tripping, slipping, and misstepping). This detailed classification will permit a more nuanced understanding of near-fall incidents, which can be used to develop targeted prevention strategies.

For the development of this classification scheme, we have already calculated several metrics related to movement and balance. These include, among others, the margin of stability, spatio-temporal components, stride duration and length, time to touchdown from perturbation (recovery performance), and acceleration and velocity for the centre of mass (CoM) and the thorax. Each of these variables will be associated with a specific threshold that will be used to determine whether a particular incident is a near-fall. By considering these variables and their thresholds, we sought to classify near-fall incidents and gain an insight into the underlying causes and dynamics of these events. These variables are as follows:

- Margin of stability (MoS): A measure of dynamic stability during walking, calculated as the difference between the extrapolated CoM and the boundary of the base of support (BoS) [61]. A lower MoS indicates a higher risk of falling [62].
- Spatio-temporal components: Parameters such as stride length, stride width, and stride duration. Changes in these parameters can indicate alterations in gait patterns, potential fall risks, and overall mobility issues [63]. Furthermore, an increase in step/stride duration or a decrease in step length may indicate an elevated risk of falls or underlying mobility issues [64]. Stride duration and stride length are utilised in fall risk assessment and in monitoring ageing-related gait instability [65]. Lastly, the time to touchdown from a perturbation (recovery performance) is a measure of the time taken by a person to take the first recovery step after a perturbation. A longer recovery time may indicate a higher risk of falling. The recovery foot’s instant of contact following a slip perturbation is an important factor [12,49]. In general, spatio-temporal components can be utilised [66,67].
- CoM velocity at touchdown: The velocity of the centre of mass of the body at the moment the foot touches the ground. A higher velocity may indicate a more unstable gait and a higher risk of falling. The velocity of the centre of mass (CoM) is utilised to characterise age-related differences and gait instability, aiding in the assessment of fall risks [68].

We plan to use these variables in the future to classify different types of incidents and their intensity.

### 2.4. Automatic STF—Classification Approach

After collecting the data and calculating ergonomic metrics, we performed preliminary classification tests using a simple decision tree model to determine the baseline performance of traditional techniques before integrating complex ML algorithms. We trained two kinds of decision trees using the Python sklearn library [69]. The first kind performs a two-class classification between baseline walking and either trips, slips, or missteps, while the second kind performs multi-class classification with all four classes. The decision tree uses the CoM and BoS measures given in Section 2.2. We projected the CoM onto the ground and measured the maximum anterior–posterior distance (in centimetres) that the CoM extended beyond the BoS and the CoM’s maximum velocity (in meters per second) for each trial before classifying it using automatically learned criteria.

Maximum CoM distance and CoM velocity were chosen as the primary kinematic parameters because of their critical role in assessing dynamic stability and balance. The speed at which a person is moving is indicated by the maximum velocity of the CoM; unusually high velocities can often be associated with increasing instability and a lack of control. The degree of imbalance is measured by the maximum distance of the CoM from the BoS; greater distances correspond to a greater risk of falling.

**Figure 2:**
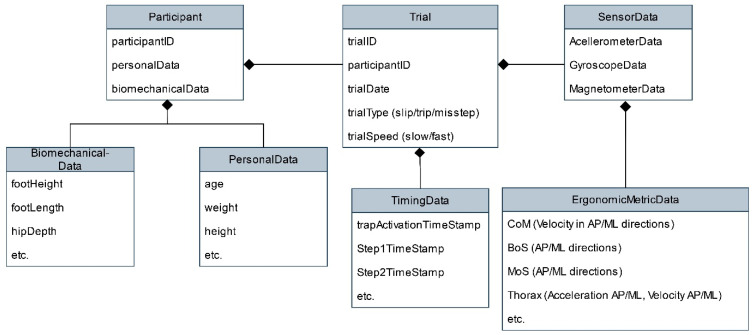
Entity–relationship diagram for the recorded data. This entity–relationship diagram shows the structure of the recorded data, how it is connected, and what kinds of data belong to the different entities. “etc.” in the attribute field indicates that the section contains example attributes only. “Biomechanical data”, for example, includes ten further biomechanical measurements.

For each classifier, half of the data was randomly selected to optimise thresholds, while the other half was used to measure its effectiveness, using accuracy, precision, and recall, F1-score, and the ROC–AUC score, as described in [70]. For all scores besides accuracy, we used a one-vs.-the-rest approach and macro averaging for the multi-class classifier, and for the binary classifiers, we treated the STF class as the positive class. We rounded scores to two digits after the decimal. For the multi-class method, we also constructed a confusion matrix (see Figure 2). We forced the two-class decision tree classifiers to maximally develop four leaf nodes which allows for potentially splitting the data once per metric. For the multi-class classifier, we restricted the decision tree to maximally develop eight leaf nodes. This prevented the decision trees from developing too many levels and overfitting the data. 

The rows of the confusion matrix indicate the actual label of a sample, while the columns indicate the expected label. Each column represents the number of samples from a particular class that were predicted to be baseline walking, stumbling, sliding, or misstepping. Thus, the diagonal cells contain the correct predictions; for instance, the top leftmost cell shows how many baseline walking samples have been correctly predicted as baseline walking. To report the trained decision trees in a compact form, we represented them as tables of the learned rules, with redundant rules being merged to minimise the tables without losing transparency of the learned decision tree thresholds. 

## 3. Results

### 3.1. Results of the Data Gathering Process

**Figure 3:**
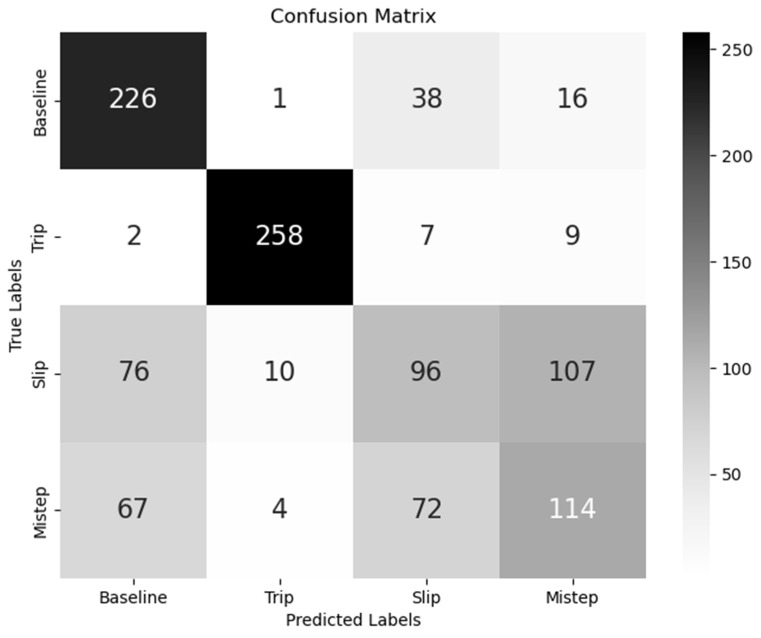
Confusion matrix showing the true labels a trial has in the rows and the predicted labels in the rows. The diagonal entries show the correct predictions.

In this project, we gathered multiple kinds of data: raw sensor data from trials and time stamps for important events like hazard activation and the steps taken, personal data (see Table 2), and biomechanical data from the participants. The dataset was further enriched with calculated ergonomic metrics. An overview of the dataset in the form of an entity–relationship diagram can be seen in Figure 3. 

Due to participants dropping out during the recording period, real falls occurring during the trials, and some participants feeling uncomfortable with certain conditions, we recorded a total of 1640 valid trials. First, considering the completeness at the trial level, the comparison between the recorded trials and the experimental design revealed the following: we initially had 110 participants and 18 conditions (recording at T1, T2, or T3; slow or fast walking speed; STF type), resulting in 1638 unique combinations of participants and conditions. This is 342 fewer than the experimental design expected. However, participants’ wishes not to perform certain conditions were respected, and some participants did not return after T1. In addition, trials in which real falls occurred were not repeated. For completeness at the sensory level, we checked all recordings for missing sensor values and found none. For metric values, we placed time stamps for steps 1 and 2 before the hazard, and steps 1 to 6 after the hazard activation. Because hazards are activated at different parts of the course—sometimes near the end—not all trials have the full six recovery step markers. In 55 trials, step 6 after the hazard was missing. In a further two files, the automatic detection of steps 3 and 5 after the hazard was incorrect, and in one case, the detection of the first step after the hazard was incorrect. In addition, one file had missing timestamps for the steps before the hazard. These problems will be corrected manually in the next version of the dataset. All other timestamps and ergonomic metrics were complete.

In total, we recorded 657 trials at T1, 633 trials at T2, and 320 trials at T3. When considering the STF type, the recorded trials were more balanced, consisting of 531 trips, 552 slips, and 557 missteps. This represents an almost perfect balance of STF types, with 32.5% trips, 33.6% slips, and 33.9% stumbles. For the walking speed, we recorded 834 trials at a slow walking speed and 806 trials at a fast walking speed. A detailed overview of the number of trials for the different STF types, speeds, and recording times can be found in Appendix A, Table A1. Following the automatic placement of the STF hazard timestamp and the subsequent manual review process to ensure correct placement, including the manual verification of the hazard name, three independent reviewers evaluated the same sample of 100 files. All three assessments confirmed that the files in this randomly selected sample were correctly labelled.

Of the 1640 test recordings, 1638 were recorded at 120 Hz and two were recorded at 240 Hz. These two files were down-sampled to 120 Hz to ensure a consistent dataset.

### 3.2. Results of the Threshold-Based STF Classification

We trained three decision trees performing binary classification and one performing multi-class classification. The scores of the classifiers can be seen in Table 3. The scores for all four different classes can be seen in Table 4. The rules learned by the decision trees can be seen in Table 5 and Table A2, Table A3 and Table A4 in Appendix A.

The accuracy results show that tripping is the most easily distinguished from baseline walking and proved successful even when all four classes are assessed concurrently (see Figure 2). In contrast, sliding and misstepping are significantly harder to differentiate, with considerably lower accuracies and substantial misclassification rates in the four-class scenario.

Interestingly, using the CoM outside of BoS-based distance metric did not improve the binary classification of baseline walking vs. misstepping, as can be seen in Table A3. Only the maximum CoM velocity remains relevant after minimising the decision tree rules, indicating that in this case, the CoM outside the BoS distance does not impact classifier performance. The decision tree rules also show that some splits in the trees were unnecessary, as can be seen especially in Table A3, whereby two leaf-nodes would have been enough for the classifier.

These findings highlight an important point: while tripping may be easily spotted using the chosen measures, the difficulty in reliably distinguishing between baseline walking, sliding, and misstepping implies limits in the threshold-based strategy. This simplistic approach does not adequately capture the complex biomechanical dynamics involved in STF occurrences. Given the rich dataset provided, which may capture subtle biomechanical nuances and temporal relationships that a threshold-based model misses, our future study will focus on more advanced AI methods. This move is not simply a step up in complexity, but a required development to overcome the limits of old methodologies. We intend to improve the precision and predictability of STF event identification and forecasting by using neuronal networks, which excel at feature discovery and event prediction in continuous data streams. This aligns with the sophisticated analytic capabilities supported by our dataset.

## 4. Discussion

This paper presents a new dataset on near-fall incidents, using data from a working population that was employed in physically demanding jobs. Our analysis of the literature reveals a significant gap; previous research has mostly involved small numbers of participants and focused on older population. Our dataset, with its larger participant base and focus on a population involved in physically demanding work, provides a sound foundation for future research aimed at automating near-fall detection and improving workplace safety. With its 17 sensors, the Xsens motion capture suit provides unparalleled biomechanical insights into near-fall events. This feature sets our dataset apart from those obtained with fewer sensors and permits a more thorough investigation of fall/near-fall mechanics. Furthermore, the nature of the dataset enables sensor-specific data to be recovered, paving the way for future research to determine the ideal, economically viable sensor array for near-fall detection. The real STF data of our dataset are a major advantage over the retrospective or simulated data often found in previous research. The increased accuracy of this method in reflecting near real-world situations makes the dataset particularly useful for building ML models—particularly neural networks—that attempt to identify falls before they occur. These algorithms have the potential to revolutionise occupational safety by providing a proactive method of fall prevention through real-time analysis and prediction, but often require more data than required for traditional ML approaches and data that are sampled from as close to the target domain as possible. With this in mind, the high number of participants and the near-real-life STF events recorded will make this dataset useful for future studies leveraging these algorithms.

Initial tests using a conventional threshold-based method have demonstrated that tripping may be quickly detected. However, extensive feature engineering and more sophisticated models are needed to distinguish between baseline walking, sliding, and misstepping. It appears more promising to apply neural networks to these problems and have them learn significant characteristics on their own, especially given the dataset’s eligibility for neural networks and their performance advantage over traditional models in many disciplines.

The dataset’s emphasis on standardised slip, trip, and misstep events may, however, limit how well it represents the range of STF scenarios that occur in different work environments. This limitation could affect how broadly ML algorithms trained with this dataset can be applied. Furthermore, the complexity of actual STF occurrences presents difficulties for data collection and classification, suggesting the need for future extensions to include a wider range of individuals, occupations, and circumstances.

It would, therefore, be beneficial for future studies to broaden the scope of this dataset by recording STF events involving more participants, participants from different fields of work and data from participants during a wider range of scenarios.

It is particularly important to categorise near-fall events according to their type and intensity, to facilitate understanding of the different risk thresholds associated with different types of near-falls. The method described here will be used to categorise the data in a future study. This could then be followed by analysis of the resulting data for creation of more targeted preventive measures by identification of specific times and movements that are more likely to lead to falls.

This study has some limitations, although it provides important information. Despite its size, the dataset may not accurately capture the variation in STF events across all work settings. Because the focus of the study was on steelworkers and parcel delivery drivers, the results may not be directly applicable to other jobs with different physical demands. In addition, the controlled setting of the tests, although necessary for standardisation, may not have fully captured the subtleties of real-world events. As this work provides a broader and more thorough dataset than previously available, its findings advance the theoretical understanding of STF events. This allows for the biomechanical elements of falls and near-falls to be analysed more thoroughly. The study also highlights the importance of including a variety of working populations in research, as this can lead to more robust and generalisable hypotheses about STF prevention.

The results of the study are important for practitioners. The information and data can be used to better target fall prevention training and programs for high-risk occupations such as parcel delivery and steel fabrication. A proactive approach to workplace safety is represented by the application of machine learning to near-fall detection, which can reduce the frequency of significant STF accidents. These findings can be used by employers and safety professionals to create safer working conditions through the implementation of more effective safety interventions and procedures.

## 5. Conclusions

The aim of this paper was to generate and analyse standardised kinematic data from STF incidents involving parcel delivery drivers and steelworkers. By addressing existing research gaps, we aimed to provide a more comprehensive dataset that reflects the diversity and demands of physically intensive jobs. This paper presents an important dataset that has the potential to revolutionise workplace safety measures against STF. Using kinematic data from real-life STF incidents involving steelworkers and parcel delivery workers, this dataset offers unprecedented insights into the dynamics of such accidents. Its reliability is underpinned by the meticulous collection process, using state-of-the-art wearable sensors to collect comprehensive multivariate time series data.

The categorisation scheme described for the classification of near-fall situations according to their intensity and type will permit a more detailed understanding of these events and their prevention. 

An outstanding feature of this dataset is its design, which is aimed at modern ML applications. The potential of these algorithms to identify individuals at a high risk of falling and to detect near-fall incidents could significantly change the landscape of fall prevention in the workplace. It has also been shown that traditional methods would need a significant amount of feature engineering to be able to tackle this complex problem—if at all. However, we expect modern ML methods to be able to learn to solve this problem to a satisfying degree. By improving the accuracy of safety protocols and enabling the development of tailored intervention strategies, modern ML techniques have the potential to usher in a new era of targeted and efficient fall prevention interventions. In essence, this dataset may not only enrich our understanding of STF events in the workplace, but also lay the groundwork for using advanced ML to promote safer working environments. Its contributions are a testament to the transformative power of integrating sophisticated data analysis techniques with occupational safety initiatives. In conclusion, this dataset is the basis for further significant contribution to the field of occupational safety. Its content will improve our knowledge of STF occurrences, but also open the door to the creation of sophisticated, data-driven preventive measures.

## 6. Outlook

The initial phase of our research will involve the application of established data quality metrics, followed by utilising the classification algorithm that is currently being developed to label the dataset using the algorithm we have describe, sorting trials into cases of strong and light near-falls. Subsequent statistical analysis will point the way to identifying the most promising ML techniques for fall prevention and detection. The use of the dataset in ML processes represents a significant step forward in our efforts to mitigate the risks associated with STF. As this research progresses, it will be necessary to develop these algorithms and rigorously test them in a range of real-world scenarios. This is essential for the full validation of their reliability and effectiveness. Challenges such as ensuring the transferability of the algorithms to different configurations and maintaining model specificity across different workplace scenarios will be critical. To enhance the robustness and applicability of our findings, future iterations of this study will aim to expand the dataset to encapsulate a more diverse range of occupations and environments. It is anticipated that such an expansion will not only increase the generalizability of our findings, but also facilitate the construction of more sophisticated ML models. In addition, exploring the incorporation of further predictive factors—ranging from cognitive to contextual characteristics—has the potential to significantly refine the accuracy of near-fall detection systems. The overall aim of this research line is to create an integrated system. This system would not only detect and predict STF incidents, but also provide personalised prevention recommendations, meticulously tailored to the unique profiles and working conditions of individual workers. The realisation of such a system holds the promise for significantly reducing the frequency and severity of workplace accidents, thereby creating safer working environments and promoting better health outcomes for workers. Subsequent research will focus on ensuring the data quality and analysing the data as outlined in this study before applying ML algorithms for the development of automatic detection and prevention measures. Further research should then expand the dataset, improving the ML methods and investigating their pragmatic application in different occupational settings.

In summary, our future efforts will not only expand the dataset and refine the ML methods, but also aim to translate these advances into practical, impactful applications for occupational safety. Through this comprehensive approach, we aim to make a significant contribution to the field, paving the way for a future in which workplace accidents are significantly reduced through the power of data-driven insights and interventions.

## Figures and Tables

**Table 1:** Overview of recent near-fall datasets in comparison to this work. The datasets were collected using body-worn equipment.

Author, Year	Number of Participants	Age	Weight	Height	Experiment Setting
This Work	110	Avg. 41.5 y (19 y–63 y)	Avg. 81.8 kg (53 kg–145 kg)	Avg. 177 cm (158 cm–202 cm)	Slips, trips, and missteps on a walkway in a laboratory setting
Rahemtulla et al. (2023) [44]	13 (13 female)	22 y–33 y	50 kg–109 kg	157 cm–172 cm	Simulating ADLs, stumbles, and falls onto a crash mat
Wang et al. (2022) [12]	34 (sex not reported)	≥60 y	-	-	Slips on a walkway in a laboratory setting
Choi et al. (2022) [43]	34 (21 male, 13 female)	Avg. 27.6 y (21 y–34 y)	Avg. 62.1 kg (45 kg–81 kg)	Avg. 167 cm (157 cm–185 cm)	Participants watched videos and simulated the activities viewed
Wang et al. (2020) [45]	10 (10 male)	Avg. 25.5 y	Avg. 78.3 kg	Avg. 176.4 cm	Descending stairs normally with a backpack, and with a backpack while simulating near-falls

**Table 2:** Overview of participants’ personal data.

	Overall Height [cm]	Height Female [cm]	Height Male [cm]	Age [years]	Age Female [years]	Age Male [years]	Weight [kg]	Weight Female [kg]	Weight Male [kg]
Average	177	168	180.6	41.5	39.5	42.3	81.8	72.1	84.9
Median	178	169	180	40	37	41	80	72	83
Min	158	158	168	19	23	19	53	53	55
Max	202	178	202	63	59	63	145	100	145

**Table 3:** Accuracy, precision, recall, and F1-scores for the different decision tree classifiers.

	Walking vs. Tripping	Walking vs. Slipping	Walking vs. Misstepping	Four-Class Classifier
Accuracy	0.99	0.76	0.77	0.63
Precision	1.0	0.77	0.77	0.61
Recall	0.98	0.74	0.76	0.61
F1-Score	0.99	0.75	0.76	0.61
ROC–AUC	0.99	0.79	0.82	0.84

**Table 4:** Precision, recall, and F1-scores of the four-class classifier calculated per class.

	Walking	Tripping	Slipping	Misstepping
Precision	0.61	0.94	0.45	0.46
Recall	0.80	0.93	0.33	0.38
F1-Score	0.69	0.93	0.38	0.42
ROC–AUC	0.88	0.97	0.74	0.77

**Table 5:** This table shows the rules learned by the four-class classification decision tree. The first columns contain the classes that are to be predicted and the second columns the rules that lead to the prediction of these classes, with v indicating the maximum velocity of the CoM and d the maximum distance the CoM left the BoS in AP directions in a trial. If a single cell contains multiple conditions, all of them have to be fulfilled for the trial to be classified as the class indicated by the tables row.

Four-Class Classification	Condition
Baseline	d < 39.07 cm v < 0.37 m/s
Trip	39.07 cm < d
	d < 39.07 cm 1.02 m/s < v
Slip	d < 39.07 cm 0.37 m/s < v < 0.45 m/s
	37.15 cm < d < 39.07 cm 0.45 m/s < v <1.02 m/s
Misstep	d < 37.15 cm 0.45 m/s < v < 1.02 m/s

## Data Availability

The data presented in this study are available on request from the corresponding author. The data are currently not publicly available, as they still need to be technically prepared for dissemination and, for legal reasons, all participating institutions must be involved in any dissemination.

## Appendix A

**Table A1:** Number of recorded trials at different recording days—T1, T2, T3—for the combinations of slow/fast STF types. Note that T1 Slow Trips and T2 Slow Missteps each contain a trial that was performed twice by the same subject.

	T1	T2	T3
Slow Trips	108	107	62
Fast Trips	102	97	55
Slow Slides	110	105	61
Fast Slides	109	107	60
Slow Missteps	109	110	62
Fast Missteps	109	107	60

**Table A2:** Representation of the decision tree differentiating between baseline walking and tripping in the form of minimised rules.

Baseline vs. Trip	Condition
Baseline	v < 0.55 m/s
	d < 30.03 cm 0.55 m/s < v < 1.43 m/s
Trip	30.03 cm < d
	d < 30.03 cm 1.43 m/s < v

**Table A3:** Representation of the decision tree differentiating between baseline walking and slipping in the form of minimised rules.

Baseline vs. Slip	Condition
Baseline	v < 0.37 m/s
	d < 20.75 cm 0.37 m/s < v < 0.45 m/s
Slip	0.45 m/s < v
	20.75 cm < d 0.37 m/s < v < 0.45 m/s

**Table A4:** Representation of the decision tree differentiating between baseline walking and misstepping in the form of minimised rules.

Baseline vs. Misstep	Condition
Baseline	0.37 m/s ≤ v
Misstep	v < 0.37 m/s
